# Active neutrophil responses counteract *Candida albicans* burn wound infection of ex vivo human skin explants

**DOI:** 10.1038/s41598-020-78387-y

**Published:** 2020-12-11

**Authors:** Christin von Müller, Fionnuala Bulman, Lysett Wagner, Daniel Rosenberger, Alessandra Marolda, Oliver Kurzai, Petra Eißmann, Ilse D. Jacobsen, Birgit Perner, Peter Hemmerich, Slavena Vylkova

**Affiliations:** 1 Septomics Research Center, Friedrich Schiller University and Leibniz Institute for Natural Product Research and Infection Biology – Hans Knöll Institute, Albert-Einstein-Str. 10, 07745 Jena, Germany; 2 Fungal Septomics, Leibniz Institute for Natural Product Research and Infection Biology – Hans Knöll Institute, Jena, Germany; 3 Institute for Hygiene and Microbiology, University of Würzburg, Würzburg, Germany; 4 Research Group Microbial Immunology, Leibniz Institute for Natural Product Research and Infection Biology - Hans Knöll Institute, Jena, Germany; 5 Center for Sepsis Control and Care (CSCC), Jena University Hospital, Jena, Germany; 6 Institute of Microbiology, Friedrich Schiller University, Jena, Germany; 7 Core Facility Imaging, Leibniz Institute on Aging – Fritz Lipmann Institute, Jena, Germany

**Keywords:** Fungal host response, Experimental models of disease, Immunology, Microbiology, Medical research

## Abstract

Burn wounds are highly susceptible sites for colonization and infection by bacteria and fungi. Large wound surface, impaired local immunity, and broad-spectrum antibiotic therapy support growth of opportunistic fungi such as *Candida albican*s, which may lead to invasive candidiasis. Currently, it remains unknown whether depressed host defenses or fungal virulence drive the progression of burn wound candidiasis. Here we established an ex vivo burn wound model, where wounds were inflicted by applying preheated soldering iron to human skin explants, resulting in highly reproducible deep second-degree burn wounds. Eschar removal by debridement allowed for deeper *C. albicans* penetration into the burned tissue associated with prominent filamentation. Active migration of resident tissue neutrophils towards the damaged tissue and release of pro-inflammatory cytokine IL-1β accompanied the burn. The neutrophil recruitment was further increased upon supplementation of the model with fresh immune cells. Wound area and depth decreased over time, indicating healing of the damaged tissue. Importantly, prominent neutrophil presence at the infected site correlated to the limited penetration of *C. albicans* into the burned tissue. Altogether, we established a reproducible burn wound model of candidiasis using ex vivo human skin explants, where immune responses actively control the progression of infection and promote tissue healing.

## Introduction

The burn wound represents a highly susceptible site for opportunistic colonization and infection by bacteria and fungi. Large wound surface, impaired local immunity, and broad-spectrum antibiotic therapy contribute to the growth of opportunistic fungal species. As a result, the incidence of fungal burn wound infections ranges between 6.3 and 44% as reported from burn centers around the world^1^, with the majority observed in patients with third degree burns^2^.

Colonization of the skin and mucosal surfaces can serve as a latent source of infection, especially in patients with impaired immune defenses^3–5^. *Candida *spp., frequent colonizers of the human skin, are the prevailing cause of fungal burn infections. Indeed, *Candida* colonization is an important risk factor for candidemia, and the risk increases substantially with the number of colonized sites. Moore et al*.* analyzed 1929 patients admitted with acute burn injury for *Candida* spp. colonization and from the 143 positive cases 43% that were colonized at more than three sites developed candidemia^5^. The development of systemic infection is due, in part, to the problematic diagnosis of candidiasis or fungal septicemia in burn wound patients due to overlapping symptoms with other illnesses such as bacteremia. Systemic candidiasis as a consequence of thermal injury has a high mortality rate of up to 74%^6^, demonstrating the potential severity of these infections.

Despite the high incidence of life-threatening *C. albicans* infections in burn wound patients, little is known about the process of infection. This, in part, is due to the lack of ethical and reliable model(s) that would allow examination of whether burn wound candidiasis is a consequence of depression of host defense mechanisms, pathogen-driven processes or other factors. Burn wound infection models currently exist for pigs^7,8^, rats^9^, and mice^10^, but have not been tested with wound-associated *Candida* spp. In general, animal models display gross differences to human skin composition, although humans and pigs share similarities in hair coat and skin structure. However, a significant limitation of the porcine model is the thickened and stiffer skin with very different biomechanical properties compared to human^11^. In addition to animal models, reconstituted human epithelia models have been employed in multiple burn wound studies^12–14^, but fail to accurately represent the complexity of the skin due to absence of features such as hair follicles, skin ducts, vascularization, and immune cell populations. The lack of immune cells makes the skin substitutes more susceptible to pathogenic colonization and infection than real skin^13^.

The use of human skin explants obtained from surgery has allowed ex vivo study of skin barrier repair^15^, wound healing^16^, chemical toxicity^17^, chronic inflammatory diseases^18^, DNA vaccination^19^, and fungal infection^20–22^. In this study we used NativeSkin skin explants to create a burn wound model of candidiasis. We have established a protocol with a high reproducibility of wounding, extended viability of the tissue and possibility for supplementation with immune cells, which allows to closely mimic the host burn wound environment. Using this model, we were able to show that debridement of the eschar leads to notable tissue penetration of *C. albicans* into the upper dermal layers. Within the course of six days the burned skin started to heal due to the active immune response to tissue damage, which included migration of resident neutrophils towards the necrotized tissue and release of pro-inflammatory cytokines. Supplementation of the model with fresh neutrophils resulted in marked infiltration of this cell type into the lower dermal layers of the skin. The active immune response limited the progression of infection. Altogether our data show that native human skin explants retain their ability to heal from burn wound induction and that active immune responses counteract *C. albicans* infection.

## Results

### Establishment of reproducible and operator independent deep second-degree burn wounds using a soldering iron

In order to establish a reproducible burn wound model, we sought standardized human skin samples that most closely represent the native skin. Therefore, we used round NativeSkin explants (Genoskin, France) obtained from surgical residues of the abdominal area of adult female volunteers. The samples have fully intact skin barrier function, stratum corneum, glands, and all cells types present in natural in vivo skin^23^. Importantly, Genoskin tests have identified NativeSkin as an immunocompetent skin model that preserves cell proliferation, including immune cell proliferation, for at least seven days of culture^23^.

**Figure 1:**
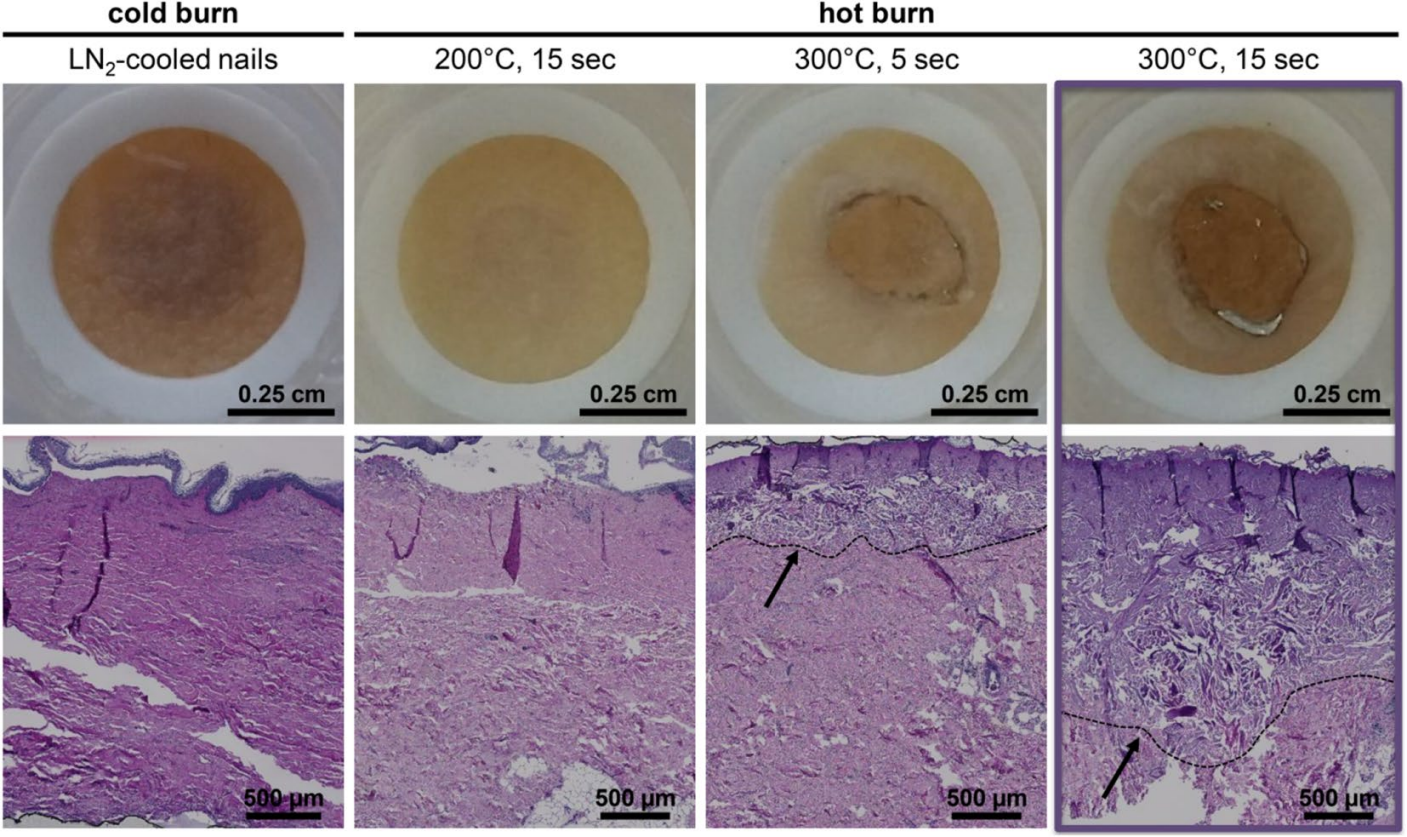
Application of a 300 °C hot soldering iron for 15 s induces deep second-degree burn wounds to human skin explants. Macroscopic (top) and microscopic PAS appearance (bottom) of skin after burn injury shows mild to profound tissue damage (burned, non-debrided, non-infected, no neutrophil supplementation). Cold burn induced by pressing liquid nitrogen-cooled brass nail for 1 min to the skin surface resulted in darkening of the skin surface and a separation of the epidermis from the dermis, inducing only a first-degree burn (day 6; N = n = 2). Hot burn was induced by using a preheated soldering iron. Only the condition of 300 °C and 15 s induced a deep second-degree burn as indicated by the disrupted collagen structure (day 1; 200 °C 15 s N = n = 1, 300 °C 5 s N = n = 1, 300 °C 15 s N = 3, n = 6). N = number of donors, n = number of technical replicates. Lines and arrows indicate the border from the intact to the disrupted collagen tissue.

Given the small area of 0.5 cm^2^ of the provided skin samples, we tested two different approaches for burn induction: cold burns were achieved by applying 2 mm head brass nails cooled in liquid nitrogen and gently pressed to the skin for 1 min; and hot burns, generated by applying a preheated soldering iron at either 200 °C for 15 s, 300 °C for 5 or 15 s (Fig. 1). The appearance of the skin was recorded after burning using the camera of a mobile phone or Stemi microscope and was compared to unburned samples.

Visually, the cold burn was signified by slight darkening of the burned area with no disruption of the skin (Fig. 1). Burning of the skin with hot soldering iron for 15 s at 200 °C led to minimal change in the color of the skin. Similar to the cold burn, no visible damage of the skin surface was observed macroscopically (Fig. 1). However, application of 300 °C to the skin resulted in prominent darkening, especially at the border of the burn, and disruption of the skin surface (Fig. 1). The macroscopic appearance of each burn type was comparable in all tested skin samples from different donors (data not shown).

**Figure 2:**
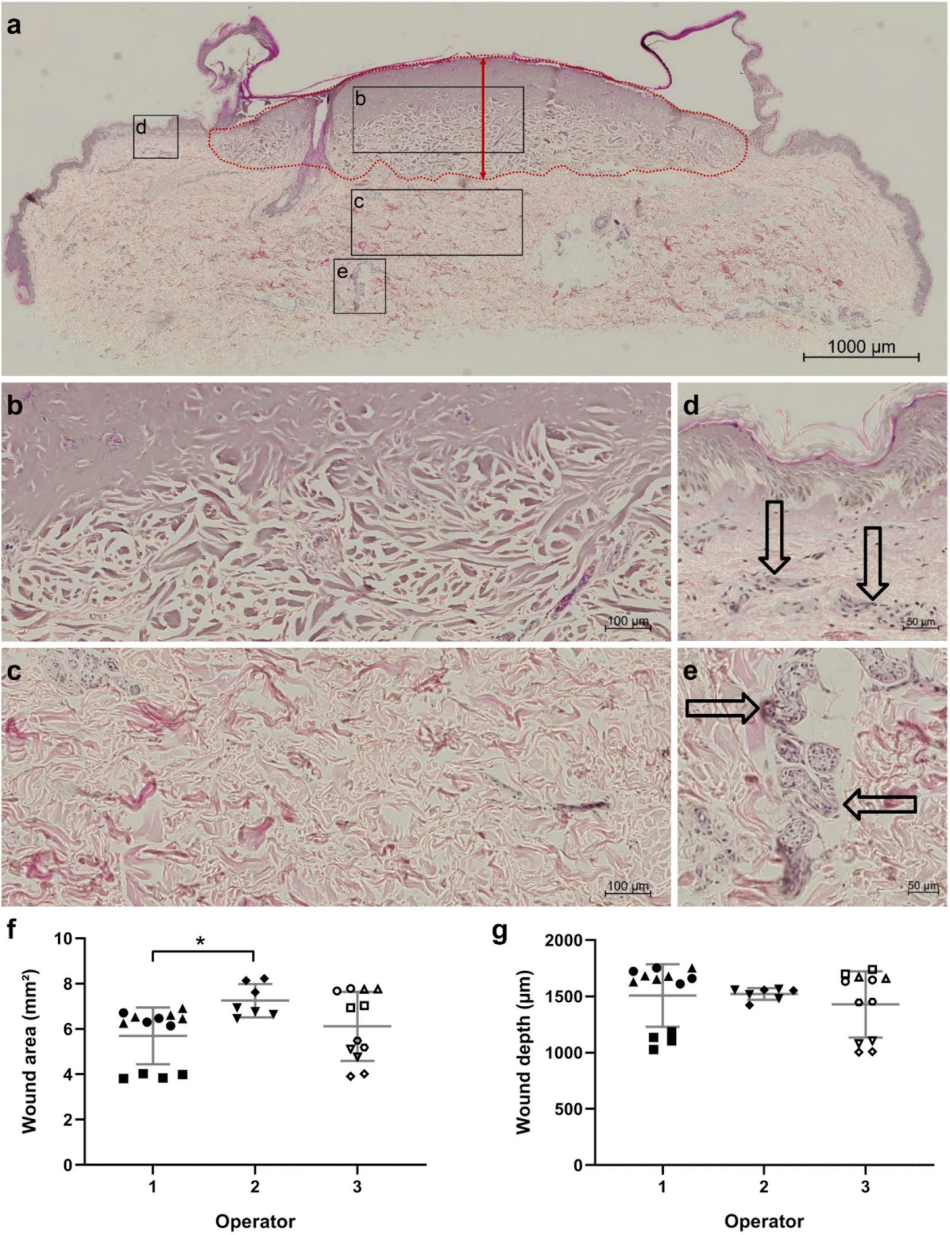
Microscopic evidence and quantification of burn injury (300 °C, 15 s). An H&E stained section of a burned skin tissue (burned, non-debrided, non-infected, no neutrophil supplementation) fixed one day post-burn wound induction ((**a**); N = 3, n = 6, representative image is shown). Cell nuclei are stained dark blue while extracellular structures are pink. Red dotted line shows the border of the burn wound as used to calculate the area of damage and the red arrow indicates the depth of the burn. Disrupted collagen structure within the burn wound (**b**) can be compared to the normal collagen structure of the lower dermis (**c**). Presence of immune cells is indicated by arrows in the upper dermis (**d**) and within glands in the subdermal tissue (**e**). Three different operators burned single skin tissue pieces ((**f**,**g**); operator 1: N = 3, operator 2: N = 2, operator 3: N = 6; black filled square, open circle etc.: each symbol represents one skin tissue piece). Multiple sections were quantified by various analyzers (operator 1: n = 13, operator 2: n = 7, operator 3: n = 12). Quantification of wound area and depth by various operators is similar as all single dots per skin sample cluster together. Burn wound induction by various operators is well reproducible as indicated by wound area (**f**) and depth (**g**), although there is a slightly larger wound area induced by operator 2. N = number of skin tissue pieces, n = number of technical replicates (skin tissue sections), One-way ANOVA with Tukey’s post-hoc test, asterisk represents significant difference between operators, *p < 0.05.

**Figure 3:**
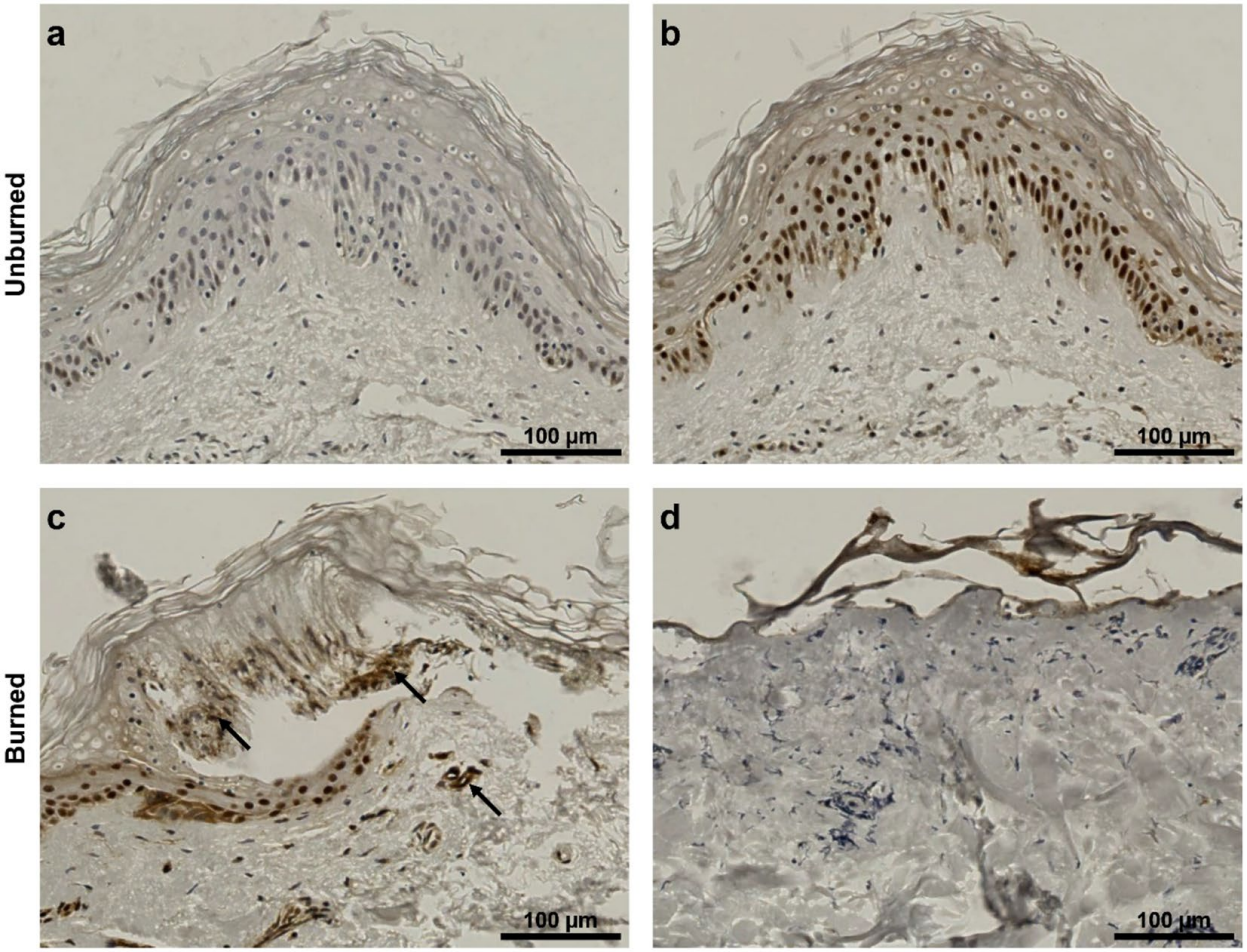
HMGB1 epithelial staining in unburned vs. burned ex vivo skin. Immunohistochemical staining of HMGB1—a marker for tissue viability. The negative control incubated without the primary antibody shows no HMGB1 background signal (**a**). In unburned tissue HMGB1 is localized to the nucleus (**b**). In burned tissue HMGB1 staining is diffuse ((**c**), black arrows) or not present anymore (**d**), showing release of HMGB1 due to cell death. Samples (non-debrided, non-infected, no neutrophil supplementation) were fixed on day 6 after burning. For number of skin donors and technical replicates see Table S1. Representative images are shown.

Histologically, thermal damage resulted in separation of the epidermis from the dermis in all cases (Figs. 1, 2a). Loss of collagen integrity in the lower dermal layers (Fig. 2a,b) was observed only in burns induced with 300 °C while burns caused by liquid nitrogen-cooled brass nails or by 200 °C soldering iron showed no to minor damage to the collagenous tissue. Therefore, cold and 200 °C-burning resulted in first-degree burns. Increasing the temperature of the soldering iron and the application time resulted in deeper burns as observed by more profound loosening of the collagenous tissue in the dermis. Importantly, the higher temperature (300 °C) markedly destroyed the dermis with the level of damage corresponding to a superficial second-degree (partial thickness) burn in the 5 s samples, and deep second-degree burn in the 15 s samples as shown by deep loss of collagen integrity in the area surrounding the burn site (Fig. 1). In addition, the detachment of the necrotized epidermis from the underlying dermis was so prominent that in some samples the epidermis was lost during preparation (Fig. 3d). Control unburned samples showed no macroscopic (data not shown) or microscopic tissue damage (Figs. S2c, S3a, S4a).

The experimental condition of soldering iron application at 300 °C for 15 s appeared to provide the targeted degree of burn damage. Therefore, we selected this setting for our further tests.

### Analyses of tissue damage following burn wound induction

To ensure reproducibility of burn wound induction by various operators, PAS or H&E stained skin sections were quantified for the area and depth of the burn wound (Fig. 2). Tissue samples were processed one day post burn wound induction. First, multiple tissue slices per skin sample were analyzed by several operators. Darker intensity of the staining (Fig. 2a) and disrupted collagen (Fig. 2b) compared to intact collagen tissue (Fig. 2c) signify the burn. This allowed us to measure the total area of damage and wound depth as scored from the apical layer to the deepest level of observed damage (Fig. 2a). All measured values per skin sample clustered together, indicating equal damage. Second, we asked three independent operators to induce burns in tissue samples from a given donor. Operator 1 burned and processed three skin tissue samples from one donor with a mean area of 5.7 ± 1.3 mm^2^ and a maximum wound depth of 1507 ± 278 µm. Operator 2 burned and processed two skin tissue samples, each from a different donor, with 7.3 ± 0.7 mm^2^ wound area and 1522 ± 53 µm wound depth. Operator 3 burned and processed six skin tissue samples, from two different donors, with 6.1 ± 1.5 mm^2^ wound area and 1429 ± 294 µm wound depth (Fig. 2f,g). We found a good correlation (Pearson r = 0.8256, p < 0.0001) of burn wound area and burn wound depth in our model. The variability between skin properties of the different donors (e.g. thickness of the skin) resulted in the highest variance. Although there was a significant difference (p = 0.0475) of the burned area, maximum wound depth was comparable. This indicates high reproducibility of the burn wound induction. A mean wound depth of 1486 µm indicates a deep second-degree burn wound—the maximum achievable depth with this model.

### Nuclear staining shows extended cell viability of the skin explants

To determine if the skin tissue remained viable throughout the experiment, the intracellular protein high mobility group box 1 (HMGB1) was stained using immunohistochemistry. HMGB1 is a nucleosome-binding chromatin factor which is released from necrotic cells as part of an anti-inflammatory response^24^. A negative control treated without the primary antibody (Fig. 3a) shows lack of background staining. The clear localization of HMGB1 to the nuclei of control samples indicates viability of cells in unburned samples after 6 days of culture (Fig. 3b). This contrasts with more diffuse staining seen at the edge of the burn wound (Fig. 3c) indicating necrosis. Furthermore, cells at the center of the burn wound were HMGB1 marker-negative (Fig. 3d), demonstrating the extreme tissue damage in this area and complete loss of individual cell structures.

### Debridement of burned skin tissue allows *C. albicans* to penetrate the dermal layer

**Figure 4:**
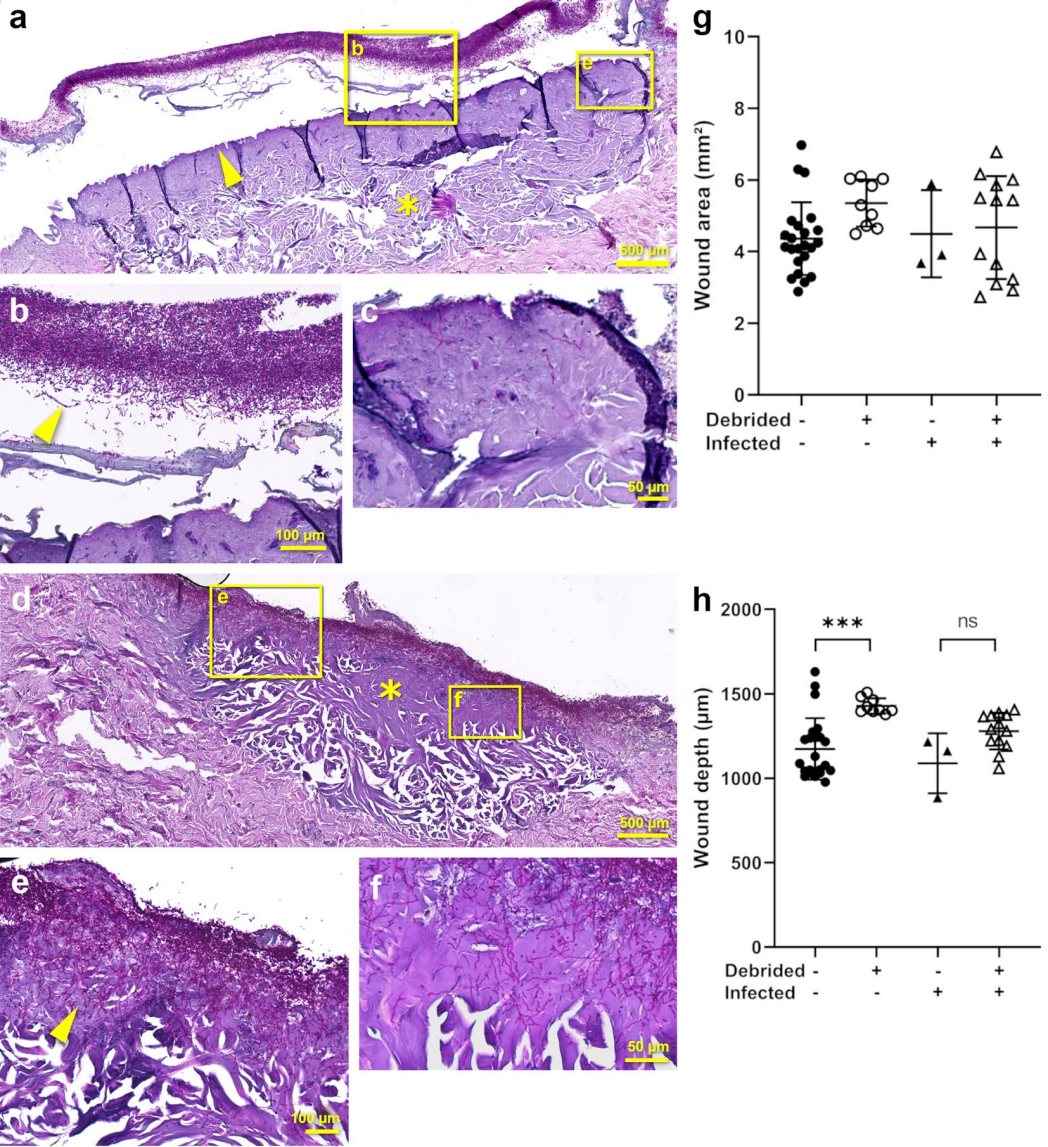
Debridement of burned skin tissue allows *C. albicans* to penetrate the dermal layer. PAS stained tissues fixed at day 6 post-burn wound induction without (**a**–**c**) and with debridement (**d**–**f**) of burns. The burned area has a darker purple color (asterisk, (**a**,**d**)) than the surrounding undamaged tissue. *C. albicans* cells stain magenta (arrows, (**b**,**e**)). Due to the burn, apical epithelial cells lose adherence and separate from the dermis (arrows,  (**a**)). *Candida albicans* is caught up at the separated epithelium in non-debrided tissues, with only few cells accessing the dermis (**b**,**c**). Following debridement, the separated epithelium is no longer present (**d**) and fungal cells invade the dermis (**e**,**f**). Fungal infection does not influence the wound area (**g**) or the wound depth (**h**). The number of skin donors and technical replicates is detailed in Table S1. Representative images are shown. Symbols are as follows: black filled triangle, black filled circle = non-debrided vs. open triangle, open circle = debrided, black filled circle, open circle = non-infected vs. black filled triangle, open triangle = infected. One-way ANOVA with Tukey’s post-hoc test; asterisks (**g**,**h**) represent significant differences between non-debrided and debrided samples, ***p < 0.001, *ns* not significant.

Once we have ensured the reproducibility of our burn wound protocol, we next proceeded with infection of the wound with *C. albicans*. The applied fungal cells accumulated mainly at the separated apical layer of the epidermis (Fig. 4a,b). Due to the physical distance to the underlying upper dermal layer, just a few cells were able to reach and penetrate the dermis (Fig. 4c).

Since removal of the eschar is considered a standard of modern burn treatment, we debrided the burn wound in order to better replicate the clinical setting. Removal of the outermost burned layer of the epithelium by debridement prior to infection allowed direct contact of fungal cells with the necrotized dermis, resulting in deeper penetration into the damaged host tissue (Fig. 4d–f). Visual comparison of the infection of non-debrided (Fig. 4a–c) and debrided burn wounds (Fig. 4d–f), reveals the difference in penetration depth. Here, notable increase in hyphal formation, a prominent virulence factor, was observed. Although debridement had no influence on the wound area (Fig. 4g), it correlated with deeper tissue damage (Fig. 4h). Fungal infection did not influence the burn wound parameters (Fig. 4h,g).

Thus, *C. albicans* can infect the burn wound and debridement of the separated epidermis allows penetration of the fungal hyphae well into the dermal layer.

### Neutrophil infiltration, cytokine activation and healing of the burned skin

**Figure 5:**
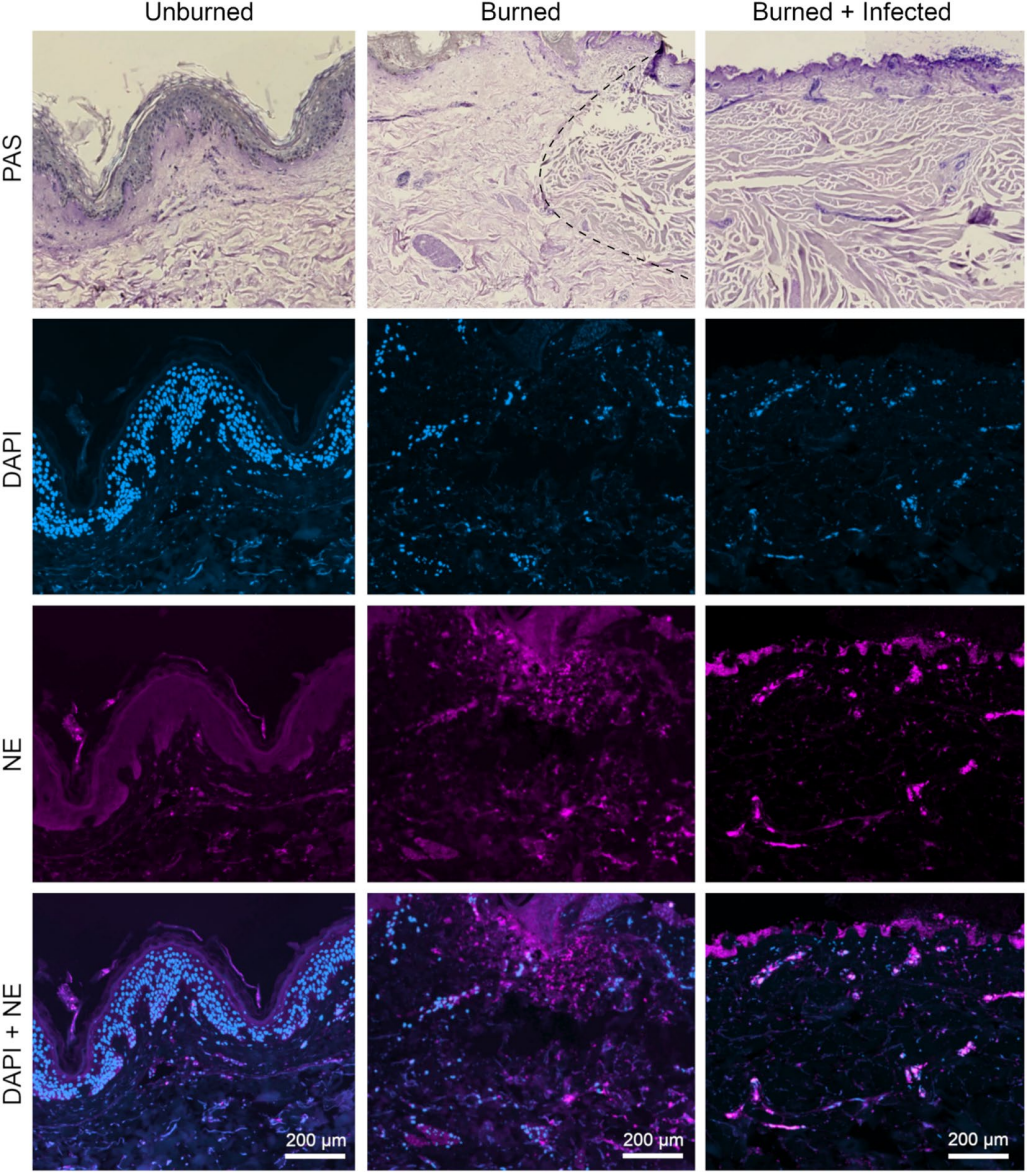
Burning of skin tissue explants attracts neutrophils to the damaged area. Unburned, burned, and burned-infected samples were stained applying PAS for a histological overview, DAPI for localization of nuclei (blue), and AF647 labelled immunofluorescence (pink) for neutrophil elastase (NE). In unburned skin neutrophils are present in the dermal layer whereas in burned and burned-infected skin neutrophils cluster near the epidermis. Defined signals of NE localize tissue-present inactive neutrophils, while diffuse signals indicate release of NE by activated neutrophils. All samples were fixed at day 6. Burn site border is indicated by the dashed line; the tissue to the right of the line is damaged. For the number of skin donors and technical replicates see Table S1. Representative images are shown. Overview slides of the corresponding samples are shown in Fig. S3.

Neutrophils are an essential part of the first line of defense that modulates repair processes following tissue damage or infection. Several pro-inflammatory cytokines, such as IL-6, IL-8, and TNF-α have been shown to be responsible for the recruitment of neutrophils to the site of infection^25–27^. Importantly, these innate immune cells play a critical role in host defense against both mucosal and disseminated candidiasis^28^. Therefore, we analyzed the presence and behavior of neutrophils in control and treated NativeSkin samples. Histological analysis of H&E staining revealed small clusters of immune cells near the vascular tissue in the upper dermis (Fig. 2d) and within the glands of the subdermal tissue (Fig. 2e). Staining with the neutrophil-specific marker neutrophil elastase (NE) showed that some of these cells are neutrophils (Figs. 5, S2a), concentrated in the vascular tissue within the dermal layers of the non-damaged skin (Figs. S2, S3d). However, larger groups of neutrophils were observed in the upper dermal layers below the epidermis of the burned samples (Figs. 5, S3e). Specifically, neutrophils migrated towards the site of injury and infection, since prominent accumulation of these cells was observed at the edge of the burn (Figs. 5, S3e,f).

**Figure 6:**
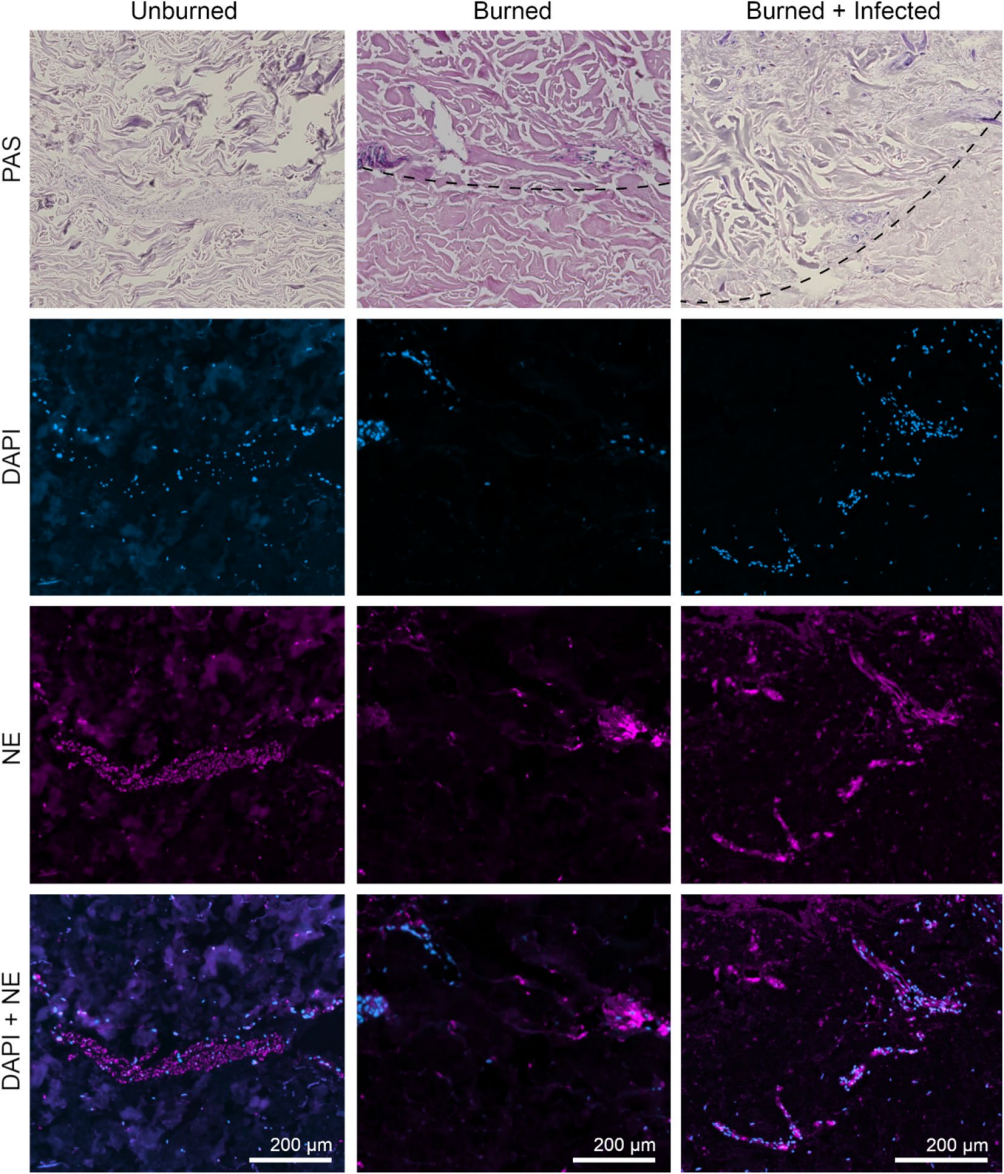
Neutrophil supplementation to the media induces wound healing of burned skin tissue explants. Unburned, burned, and burned-infected samples were stained applying PAS for a histological overview, DAPI for localization of nuclei (blue), and AF647 labelled immunofluorescence for neutrophil elastase (NE, pink). Following neutrophil supplementation larger clusters of neutrophils are present in the lower dermal layer of unburned skin tissue. Defined signals of NE localize tissue-present inactive neutrophils, while diffuse signals indicate release of NE by activated neutrophils. Due to burning or burning and infection neutrophils migrate towards the upper dermal layers and cluster at the site of damage and infection. All samples were fixed at day 6. Burn site border is indicated by the dashed line; above the line the tissue is damaged. For the number of skin donors and technical replicates see Table S1. Representative images are shown. Overview slides of the corresponding samples are shown in Fig. S4.

Neutrophils migrated towards the site of injury (Figs. 5, S3), but the number of neutrophils present in the system was limited by the absence of blood supply. Therefore, we investigated the effect of neutrophil supplementation on the model (Figs. 6, S4). We applied freshly isolated human immune cells to the growth medium on the basal side of the tissue and analyzed neutrophil infiltration at day 6 post infection. Immune cell recruitment to the lower dermal layer was already notable in the unburned samples (Figs. 6, S4d). Damage of the skin by induction of deep second-degree burns resulted in stronger infiltration of the neutrophils to the upper dermal layers of the tissues (Figs. 6, S4e), which was further increased in *C. albicans*-infected burned samples (Figs. 6, S4f). Altogether, we were able to verify the presence of resident neutrophils in the skin samples and showed that these cells become recruited to the damaged tissue in response to wounding and *C. albicans* infection.

**Figure 7:**
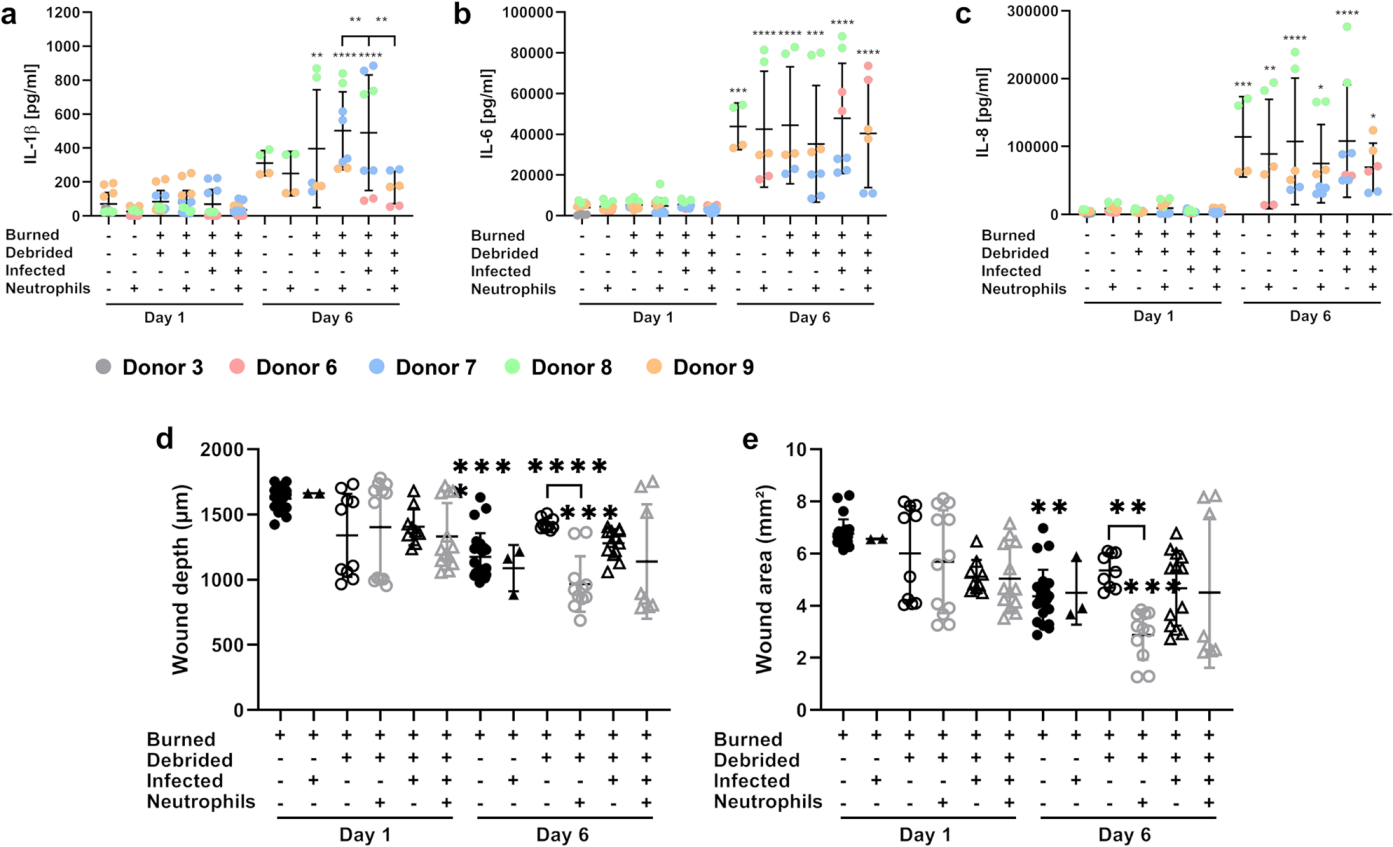
Wound area and depth are decreasing over time paralleled by progressing cytokine release. From day 1 to day 6 cytokine release increases over time (**a**–**c**), whereas wound area (**d**) and depth (**e**) tend to decrease. For IL-1β, debridement further increases cytokine release (**a**) whereas for IL-6 and IL-8 debridement has no influence (**b**,**c**). For IL-6 and IL-8, there is a tendency towards reduced cytokine release following neutrophil supplementation although this difference is not significant (**b**,**c**). If skin tissues are infected, debrided or both, neutrophil supplementation partially reduces wound area (**d**) and wound depth (**e**). For number of skin donors and technical replicates see Table S1. Colors represent various donors (**a**–**c**). Symbols are as follows: filled triangle, filled circle = non-debrided vs. open triangle, open circle = debrided, filled circle, open circle = non-infected vs. filled triangle, open triangle = infected, black = without neutrophil supplementation vs. grey with neutrophil supplementation (**d**,**e**). One-way ANOVA with Tukey’s post-hoc test testing day 1 vs. day 6 (asterisks without brackets) and without vs. with neutrophils (asterisks with brackets). Levels of significance: *p < 0.05, **p < 0.01, ***p < 0.001, ****p < 0.0001.

To further characterize the host response, we measured the release of the pro-inflammatory cytokines IL-1β, IL-6, and IL-8 at day 6 post burn induction and compared it to day 1 (Fig. 7a–c). Release of all three cytokines was noted in control, burned, and burned-infected skin either with or without neutrophil supplementation, at both time points. Moreover, we found major differences between results from each donor. Under most conditions, levels of all cytokines were significantly increased from day 1 to day 6 (~ fivefold for IL-1β; ~ ninefold for IL-6, ~ 18-fold for IL-8), although IL-1β was released at a much lower level than IL-6 or IL-8. For IL-1β, there was a trend of increased cytokine release due to burning or burning and infection compared to unburned control samples at day 6 (Fig. 7a). For IL-6 and IL-8, burning and infection with *C. albicans* had no influence on cytokine release compared to unburned controls indicating that aging of the tissue during the experimental time frame alone leads to increased cytokine release (Fig. 7b,c).

Comparing IL-6 and IL-8 levels on day 6, we noted that neutrophil supplementation led to a tendency towards decreased cytokine release compared to non-supplemented samples. As neutrophil supplementation supports tissue healing, we also asked whether the burned skin tissue was healing over time. To this end, we analyzed wound area and wound depth from day 1 and day 6 samples (Fig. 7d,e). At the later time point, there was a significant decrease of wound area and depth of the burned samples, especially following neutrophil supplementation (Fig. 7d,e). Similar trend was noted in the infected samples, though the effect was not significant. Therefore, the damaged skin tissue is healing over time.

## Discussion

Animal models have been indispensable for biological research as working model systems to investigate human diseases. However, in recent years the ethical issues surrounding the exploitation of animals and mistreatment in areas such as cosmetic research has been severely questioned. A surrogate for human skin is a reconstituted human epithelium, but this model has the disadvantage of lacking the complex structure of real skin. Therefore, the use of human skin explants such as NativeSkin as an ex vivo alternative to animal models, with a close resemblance to the host environment, are gaining popularity. Similar ex vivo models of skin explants were used to study other methods of damage and their influence on fungal infection, such as abrasion^29^, UV damage^30^, and laser damage^31^.

### Establishment and reproducibility of an ex vivo human skin burn wound infection model

The goal of this study was to establish a reproducible model of burn wound infection. Using a soldering iron with a set temperature we managed to create deep second-degree burns in human skin explants. Deep second-degree burn wounds are characterized by damage of the epidermis and large areas of the dermis, but underlying fat tissue, nerves, and muscles are not affected^32^. Throughout this study in total 39 skin tissue samples from six different donors were burned at 300 °C for 15 s by multiple operators. Although burn size and depth were comparable between tissue pieces, we noted variation between donors. This could be considered as an advantage of the use of skin explants, as it gives the chance to monitor cutaneous responses of different skin types and therefore gain a better understanding of individual variability. Previous reports on the use of skin explants in disease research found individual variability among skin donors, including thickness of the skin, distribution of cell types and differences in immune and other responses^33,34^. In this work, the six skin donors were all female, Caucasian and with skin type 2 or 3 leading to differences in skin color corresponding to the Fitz Patrick classification scale^35^. The inflicted burns were similar between different operators, validating the reliability and reproducibility of the model (Fig. 2f,g). Major differences between results from each donor were found especially for cytokine responses (Fig. 7). Therefore, the burn wound model developed herein can reflect the patient variability found in the clinic.

A further requirement of the model is that tissue must remain viable throughout the duration of the experimental time frame. Previous studies have reported high apoptosis rates in skin explants after 5–6 days incubation at 37 °C^36^. In this work cell viability was confirmed by HMGB1 staining of nuclei in skin tissues after 6 days, when not affected by burning (Fig. 3). Due to manufacturer’s guidelines tissues were not analyzed beyond this time point. Other human skin explant models have reported retained viability and proliferative capacity of cells for up to 14 days at 37 °C, when supplemented with media containing 10% fetal calf serum (FCS)^22^. Although addition of FCS to the media could also be tested for the effect on viability, this could potentially stimulate hyphal growth of *C. albicans* and therefore affect the outcome of infection. Quantitative measurements of the burn wound showed that both depth and area of the burn were reduced over time from day 1 to day 6 (Fig. 7), indicating healing potential of the tissue. This is another aspect of the model that simulates in vivo host conditions.

The human skin is a reservoir of the commensal fungus *C. albicans* and superficial fungal infections can typically originate from an individual’s endogenous microflora. Major disruption of the skin barrier and creation of a favorable environment for growth, as it may occur in a severe burn wound, can promote a switch of *C. albicans* from commensal to pathogen and lead to systemic infection. In clinics, debridement of the eschar is a common method for treatment of burn wounds^37^. By removing this layer of tissue, debridement resulted in a higher proportion of *C. albicans* attachment to and penetration of the dermis (Fig. 4) in our model. However, debridement was not observed to influence host responses, such as cytokine release or neutrophil recruitment. Yet, the skin explants managed to control the infection, suggesting that, at least in this model, *C. albicans* alone is not a significant source of infection of burned immunocompetent skin. The healing potential was somewhat reduced in the infected skin samples. Such open wounds could be vulnerable to secondary infections by, for example, bacterial skin pathogens. Thus, our burn wound infection model could be used to further investigate the correlation between burn wound debridement and the development of infection, the effect of secondary infections or treatment therapies.

### Immune responses

Burn injury typically suppresses cell-mediated immunity^38^, therefore it was interesting to evaluate the extent of this response in burned skin explants. Genoskin has previously demonstrated immunostaining for the markers CD207 (Langerhans cell), CD3 (skin resident T cell), HLA-DR (dendritic cell), and tryptase (mast cell) positive populations present in NativeSkin, but not for neutrophils. Unlike lymphocytes and dendritic cells, neutrophils are not abundant in healthy skin, but are recruited from the bloodstream or lymphatic vessels to populate the skin in inflammatory conditions or after wounding^39^. In our model staining of the unburned skin tissue for neutrophil elastase, concentrated in the nuclei of neutrophils and neutrophil precursors, show presence of neutrophils in the upper layers of the dermis (Figs. 5, S2). Burning and infection of the skin caused these cells to gather around the edges of the burn wound. This suggests that the limited number of resident neutrophils were actively recruited to the site of the tissue damage.

In order to mimic the vascular system of the host environment more closely, the burn wound model was supplemented with freshly isolated neutrophils, added to the culture medium at the initiation of infection (Figs. 6, S4). The occurrence of neutrophils in the subdermal tissue was observed only after supplementation, which is a clear indication for migration from media to tissue. It should be noted that this response also occurred to some extent in the absence of wounding or infection. The presence of large groups of neutrophils in the epithelial tissue near the edge of the burn wound may be interpreted as a result of specific immune recruitment due to injury.

Our model allows further analyses of neutrophil recruitment and immune cell signaling. For example, it has been shown that neutrophils induce upregulation of TLR4 in oral epithelial cells through secretion of cytokine TNF-α, potentiating fungal recognition and tissue protection^40^. Measuring expression of TLR4 or TNF-α in the presence or absence of neutrophil supplementation to this skin model could determine if neutrophils induce similar changes in the skin. Furthermore, the role of neutrophils in epithelial antifungal defense has been reported to be tissue specific^41^ and therefore it will be interesting to characterize this host response in the context of a skin-specific burn wound environment. A protective anti-*Candida* Th1-type epithelial response is likely to contribute to the recruitment of neutrophils to the site of infection^14^.

Further characterization of the immune responses to thermal injury showed that after 1 day of burning and infection the released cytokine levels were rather low, but after 6 days all measured cytokines increased. For IL-1β, burning resulted in a slight increase of the cytokine response. However, infection with *C. albicans* had no effect on cytokine production. The levels of IL-6 and IL-8 increased in unburned uninfected samples at day 6, which suggests that aging of the tissue also leads to production of these cytokines, despite the lack of a wound or burn stimuli. In contrast, due to neutrophil supplementation cytokine levels tend to decrease for IL-6 and IL-8 for all conditions at day 6. IL-8 is known to be involved in neutrophil recruitment^25^ and IL-8 gene expression is increased when ex vivo skin tissues are infected with *Trichophyton rubrum,* showing signaling for recruitment of immune cells to the damaged tissue^22^. Therefore, decreased IL-8 levels seem to be contradictory. We speculate that increased neutrophil tissue infiltration following supplementation of the media results in lower stimulation of cytokine release from the host cells.

### Burn wound candidiasis

This work specifically analyses *C. albicans* infections. Infections by other *Candida* species will be modelled in the future considering the trend towards increase of non-*albicans* species infections in burns and ICU patients^42^. This may be of particular importance as the outcome for septic patients infected with *Candida glabrata* and *Candida tropicalis* appears to be worse than for *C. albicans*^38^. Moreover, *Candida auris* infections are becoming increasingly important, also in the context of burns^43^.

Finally, mixed infections of microorganisms such as interactions between bacteria and fungi within the burn wound are varied and complex, often contributing to clinical pathogenesis of infection. An example of such an interaction is that presence of *Pseudomonas* sp. in the burn wound (isolated from 41% of burn wound patients included in the study) significantly inhibits growth of *Candida *sp. also in the burn wound^44^. Analysis of such complex infections may give insights into infection processes and virulence factors and might be one step further in the development of treatment strategies for burn patients.

## Conclusion

In this work a native human burn wound infection model of candidiasis has been established. Human skin explants were wounded by burning in order to study the progression of fungal infection by *C. albicans* WT SC5314. The method achieves high consistency and reproducibility of wounding. Such an ex vivo model is a step away from traditional animal models, as advocated for by the National Centre for the Replacement, Refinement & Reduction of Animals in Research (NC3R). Having established a model system with an active population of neutrophils allows further analyses of neutrophil behavior, immune signaling, and tissue healing. Also, other immune cells, which are resident in human skin like Langerhans cells or mast cells, can be addressed in order to understand their role in burn injuries and secondary infections. In general, the model offers the investigation of different types of skin trauma like scratches and stiches, also in context of infection. The processes of skin infections of all kinds of pathogens and the way how the virulence factors contribute to the infection can be studied as well, also for mixed infections which are common, not only in burn wounds. Furthermore, applying a native ex vivo skin infection model enables the investigation of the effect of potential topical therapeutics against skin infections and wound healing.

## Materials and methods

### Human skin explants

Human skin model NativeSkin was used in this study (Genoskin, France) (Fig. S1). The skin donors were healthy 30–61 years old female Caucasians that underwent abdominoplasty. None of the skin donors suffered from skin disease, although some had allergies (Table 1).

**Table 1:** Characteristics of skin donors used in this study.

Donor	Age (years)	Skin type (Ref.^35^)	Size (cm)	Weight (kg)	Allergies	Treatment
2	61	2	157	75	Ofloxacin	None
3	45	2	173	85	None	None
4	47	2	160	80	None	None
5	47	3	164	64	None	None
6	31	2	164	88	Latex	Codein, Alprazolam, Vitamins
7	30	3	150	71	None	None
8	38	2	157	65	None	None
9	36	2	166	54	Iodine, Penicillin	None

### Candida albicans strain and media

*Candida albicans* strain SC5314 was used in this study. The strain was cultivated on YPD agar plates (Yeast extract Peptone Dextrose, 20 g/l peptone, 10 g/l yeast extract, 20 g/l glucose, 20 g/l agar–agar, pH 5; Carl Roth, Germany) for two days at 30 °C. For overnight (ON) cultures, cells were grown in liquid YPD (20 g/l glucose, 20 g/l peptone, 10 g/l yeast extract; Carl Roth, Germany). For log growth cultures, the ON culture was adjusted to an optical density of 600 nm (OD_600_) of 0.2 in 10 ml of liquid YPD and incubated for 4 h at 37 °C, 180 rpm.

### Burn wound induction and debridement of ex vivo human skin

Twelve equally sized 0.5 cm^2^ skin samples per donor were delivered less than 48 h after surgery. Upon delivery the skin samples were allowed to equilibrate to 37 °C and 5% CO_2_ for 1 h. Cold burns were induced by applying 2 mm head brass nails cooled for 1 min in liquid nitrogen and gently pressed to the skin for another 1 min. Hot burn wounds were induced using a 100 W LS-100D II soldering iron (ELV Elektronik AG, Germany) with a 45° angled tip (ELV Elektronik AG, Germany) preheated to 200 °C or 300 °C. The device tip (0.08 cm^2^) was held for 5 or 15 s in an upright, angled position to allow direct contact with the skin surface. No additional pressure was applied by the operator. In some instances, a sterile pipette tip was used to break the contact between the skin and the tip of the soldering iron. Following the burn, the tip of the soldering iron was wiped with steel wool to remove any debris. The tissues were debrided by scraping the surface of the wound with the blade of a scalpel (Carl Roth, Germany) followed by gentle washing with 20 µl PBS to remove any debris. The burned skin samples were incubated for 2 h at 37 °C prior to infection.

### Burn wound infection

To infect the burn wounds, logarithmic phase culture of *C. albicans* SC5314 strain was washed, counted, and adjusted to 1 × 10^7^ cells/ml in PBS. Ten microliter of this suspension was added to the center of the burn wound resulting in an infectious dose of 1 × 10^5^ cells per wound.

### Neutrophil isolation and supplementation

Neutrophil isolation from whole human blood was done using MACSxpress Neutrophil Isolation Cocktail Kit (Miltenyi Biotec B.V. & Co. KG, Germany) according to the manufacturer’s instructions. Isolated cells were counted using Auto Hematology Analyzer BC-5300 (Mindray Medical Germany GmbH, Germany).

100 µl of isolated cells were mixed with antibodies against CD66b (neutrophils; 2 µl 1:4 diluted, Pacific blue-labelled; BioLegend, CA, USA), CD14 (monocytes; 1 µl undiluted, PerCP-Vio700-labelled; Miltenyi Biotec B.V. & Co. KG, Germany), CD3 (lymphocytes; 1 µl undiluted, VioGreen-labelled; Miltenyi Biotec B.V. & Co. KG, Germany) and CD45 (immune cells; 1 µl undiluted, PE-labelled; Miltenyi Biotec B.V. & Co. KG, Germany). After 10 min incubation at 4 °C, stained cells were washed with 1 ml cell wash by centrifugation for 5 min at room temperature (RT) and 300×*g*. The cell pellet was resuspended in 100 µl cell wash to analyze purity of the isolated cell fraction using FACS (BD FACSCanto II; BD Biosciences, San Jose, CA, USA). Using FACS analysis, we found that about 96–98% of cells were positive for CD66b indicating a very high amount of neutrophils in the obtained cells (data not shown). 2 × 10^6^ cells in RPMI with 5% heat-inactivated human serum (50 µl) were added to the surrounding medium of the skin samples.

### Skin processing and staining

The skin samples were incubated at 37 °C and 5% CO_2_ and processed on day 1 and 6 post-infection. Prior to processing the macroscopic appearance of tissues was recorded with a mobile phone or a Stemi 2000-C Stereo Microscope (Carl Zeiss Microscopy GmbH, Germany). Next, the medium surrounding the skin inlet was removed from each well and aliquots of 300 µl were immediately frozen in liquid nitrogen, and stored at − 80 °C.

Skin tissues were removed from the plastic insert and transferred to the lid of a polystyrene petri dish for dissection. Curved metal forceps and a Cutfix disposable scalpel with a curved blade (B. Braun, Germany) were used to bisect the tissue. One half was transferred to RNAlater (invitrogen, ThermoFisher, Germany), snap frozen in liquid nitrogen, and stored at − 80 °C in RNase-free tubes for future analysis. The other half was fixed in Roti-Histofix 4% formaldehyde solution (Carl Roth, Germany) for one week. The fixed tissues were embedded in paraffin blocks using Shandon Citadel 1000 (Thermo Scientific, Germany), cut into 4 µm sections with HM355S Automatic Microtome (ThermoFisher, Germany), and the sections attached to SuperFrost Plus Gold slides (ThermoFisher, Germany). Hematoxylin and eosin (H&E) and periodic acid-schiff (PAS) staining were done using H&E fast staining kit and PAS staining kit (Carl Roth, Germany), respectively, according to the manufacturer’s instructions, mounted on slides with Roti-Histokitt II (Carl Roth, Germany), and dried overnight.

### Assessment of the burned area, wound depth, and tissue injury

The prepared tissue slices were imaged in full using either the Biotek Cytation 5 (BioTek Instruments GmbH, Germany) or the slide scanner Axio Scan.Z1 (Carl Zeiss Microscopy GmbH, Germany) and used for quantitative measurements of the maximum depth and total area of the burn damage with ImageJ v1.512 software^23^ or Zen v3.1 (blue edition; Carl Zeiss Microscopy GmbH, Germany) software. The transmission images were recorded with a 10× objective. Markers of tissue injury, e.g. presence of inflammatory cells and disrupted collagen structure, were identified from fully imaged tissue slices in H&E stained sections by light microscopy. Multiple slices per skin sample were measured for wound area and wound depth by multiple operators (in total 129 values). Skin tissue sections that could not be accurately measured due to separation of the dermis and epidermis or ruptures in tissue structure occurring during processing and sectioning were excluded from the analysis.

### Immunohistochemical staining for HMGB1

Immunohistochemical staining for HMGB1 was performed as previously published^45^ with the following modifications: Endogenous peroxidase activity was blocked by incubating sections in 3% H_2_O_2_ for 10 min at RT. Antigen retrieval was performed in sodium citrate buffer (10 mM sodium citrate, 0.05% Tween 20, pH 6.0) for 10 min at 90 °C using a pressure cooker Decloaking Chamber NxGen (Biocare Medical, USA). Samples were slowly cooled down for 45 min at RT. Nonspecific binding sites were blocked with 10% normal goat serum in 1% BSA in 1× Tris-CSA buffer (50 mM Tris, 300 mM NaCl, 0.001% Tween 20, pH 7.6). Tissue sections were incubated with the primary antibody anti-HMGB1 (ab18256; Abcam, UK), diluted 1:1000 in 1% BSA in 1× Tris-CSA buffer, and incubated overnight at 4 °C. Sections were washed five times in 1× Tris-CSA and incubated for 30 min at RT with biotinylated goat anti-rabbit IgG secondary antibody (BA-1000; Vector Laboratories, USA), diluted 1:200 in 1% BSA in 1× Tris-CSA. After washing with 1× Tris-CSA, slides were stained using the VECTASTAIN Elite ABC-HRP Kit (Peroxidase, Standard; Vector Laboratories, USA) according to the manufacturer’s instructions and 3,3-diaminobenzidine (DAB) (Vector Laboratories, USA) as substrate. The reaction was stopped after 40 s using distilled water. Finally, the sections were counterstained in hemalaun (Carl Roth, Germany) for 10 min at RT, washed for 5 min under running tap water for blueing, dehydrated using an increasing ethanol row, and mounted with Roti-Histokitt II (Carl Roth, Germany). Whole immunohistochemistry images were scanned using Axio Scan.Z1 with a 10× objective (Carl Zeiss Microscopy GmbH, Germany). Images were prepared using Zen3.1 (blue edition; Carl Zeiss Microscopy GmbH, Germany) software. Negative controls were incubated with 1% BSA in 1× Tris-CSA without the primary antibody.

### Immunofluorescent staining for NE

Slides were processed for immunostaining with anti-NE antibody (ab131260; Abcam, UK), diluted 1:100 in antibody dilution buffer (1% BSA and 0.5% Triton X-100 in TBS-T) and incubated overnight at 4 °C as follows: deparaffinization by a series of washes in xylol (3 × 10 min), 100% ethanol (2 × 5 min), 96% ethanol (2 × 5 min), and 70% ethanol (1 × 5 min); permeabilization (1 × 10 min in 0.2% Triton X-100 in freshly prepared TBS-T buffer); antigen retrieval (Tris/EDTA buffer: 10 mM Tris, 1 mM EDTA solution, 0.05% Tween 20, pH 9.0) at 95 °C for 10 min using the Decloaking Chamber NxGen (Biocare Medical, USA); cool down slowly; washing in TBS-T (3 × 10 min), and blocking for 1 h in 5% normal goat serum in TBS-T (ThermoFisher, Germany). After incubation with the primary antibody, slides were washed in TBS-T (3 × 10 min). As a secondary antibody AlexaFluor 647-labeled goat anti-rabbit IgG (ab150079; Abcam, UK) diluted 1:300 in antibody dilution buffer was used for 2 h at room temperature in the dark. Slides were washed in TBS-T (3 × 10 min) and left to dry for 5 min. Mounting was done using ProLong Diamond Antifade Mountant with DAPI (ThermoFisher, Germany) overnight and imaged using the Axio Scan.Z1 with a 20× objective (Carl Zeiss Microscopy GmbH, Germany). Z-stack imaging of skin tissue slices was performed by using a Axio Scan.Z1 (Carl Zeiss Microscopy GmbH, Germany) equipped with a Colibri LED light source, a color camera (HV-F202SCL, Hitachi Kokusai Electric Inc., Japan) for brightfield imaging, and a monochrome camera (Axiocam 506 mono, Carl Zeiss Microscopy GmbH, Germany) for fluorescence imaging. The brightfield stacks were acquired with a 10× objective (Plan-Apochromat, 10×/0.45, Carl Zeiss Microscopy GmbH, Germany) and the fluorescence stacks with a 20× objective (Plan-Apochromat, 20×/0.8, Carl Zeiss Microscopy GmbH, Germany). To build 2D extended depth of focus images the stacks were processed with the ZEN v2.3 software (Carl Zeiss Microscopy GmbH, Germany). Samples incubated with antibody dilution buffer without the primary antibody were used as a control.

### Milliplex immunoassay

The MILLIPLEX MAP Kit—Human High Sensitivity T Cell Magnetic Bead Panel (Merck KGaA, Deutschland) was used to detect levels of the cytokines IL-1β, IL-6, and IL-8 in media collected from the native skin model stored at − 80 °C after freezing in liquid nitrogen. Media samples were taken from day 1 and day 6 (no media exchange between day 2 and 6). The assay was carried out according to the manufacturer’s instructions with incubation steps using an Eppendorf ThermoMixer comfort shaker (Eppendorf AG, Deutschland) at 550 rpm. Measurement of the plate and data analysis were done using Bio-Plex 200 System (Bio-Rad Laboratories GmbH, Germany), according to the manufacturer’s instructions. The optimal dilutions for the concentration of each cytokine were 1:1000 for IL-6 and IL-8, while 1:2 dilution was used for IL-1β.

### Human samples

The collection, manufacture, and use of skin models (NativeSkin) for research purposes were formally authorized by the French Ministry of Research (AC-2017-2897, 12 Oct 2017) and approved by the French Ethical Committee (Comité de Protection des Personnes (CPP)). Written informed consent was obtained from all participants. According to European and national regulations, the use of this commercially available model does not require additional ethics reviews.

Human peripheral blood was collected from healthy volunteers with written informed consent. This study was conducted in accordance with the Declaration of Helsinki and all protocols were approved by the Ethics Committee of the University Hospital Jena (permit number: 273-12/09).

### Statistics

In this work “N” corresponds to the number of biological replicates (skin tissue pieces (Fig. 2) or skin donors (all other figures)) and “n” to the number of technical replicates (see Table S1 or figure legends). Statistical analyses were done using GraphPad Prism v8 (GraphPad Software, USA). The data are expressed as mean ± standard deviation (SD). ROUT outlier test (Q = 1%) was performed if more than four technical replicates were available. One-way ANOVA with Tukey’s post-hoc test was used to compare each pair of data set in order to establish significance. Statistically significant p values are defined as follows: *p < 0.05; **p < 0.01; ***p < 0.001; ****p < 0.0001.

## Supplementary Information

Supplementary Information.
