# Supplementary material for: Active neutrophil responses counteract Candida albicans burn wound infection of ex vivo human skin explants
Source: Sci Rep. 2020 Dec 11;10:21818. doi: 10.1038/s41598-020-78387-y (PMC7732850; doi:10.1038/s41598-020-78387-y)
Supplement: Supplementary file 1 — Supplementary Information. [file 41598_2020_78387_MOESM1_ESM.pdf]

Active neutrophil responses counteract *Candida albicans* burn wound infection of *ex vivo* human skin explants

Christin von Müller[1*], Fionnuala Bulman[1*], Lysett Wagner [1], Daniel Rosenberger[1], Alessandra Marolda[2], Oliver Kurzai[2,3], Petra Eißmann[4], Ilse D. Jacobsen[4,5,6], Birgit Perner[7], Peter Hemmerich[7], Slavena Vylkova[1*]

[1]Septomics Research Center, Friedrich Schiller University and Leibniz Institute for Natural Product Research and Infection Biology - Hans Knöll Institute, Jena, Germany

[2]Fungal Septomics, Leibniz Institute for Natural Product Research and Infection Biology - Hans Knöll Institute, Jena, Germany

[3]Institute for Hygiene and Microbiology, University of Würzburg, Würzburg, Germany.

[4]Research Group Microbial Immunology, Leibniz Institute for Natural Product Research and Infection Biology - Hans Knöll Institute, Jena, Germany

[5]Center for Sepsis Control and Care (CSCC), Jena University Hospital, Jena, Germany

[6]Institute of Microbiology, Friedrich Schiller University, Jena, Germany

[7]Core Facility Imaging, Leibniz Institute on Aging - Fritz Lipmann Institute, Jena, Germany

[*]Authors share co-first authorship.

Supporting information

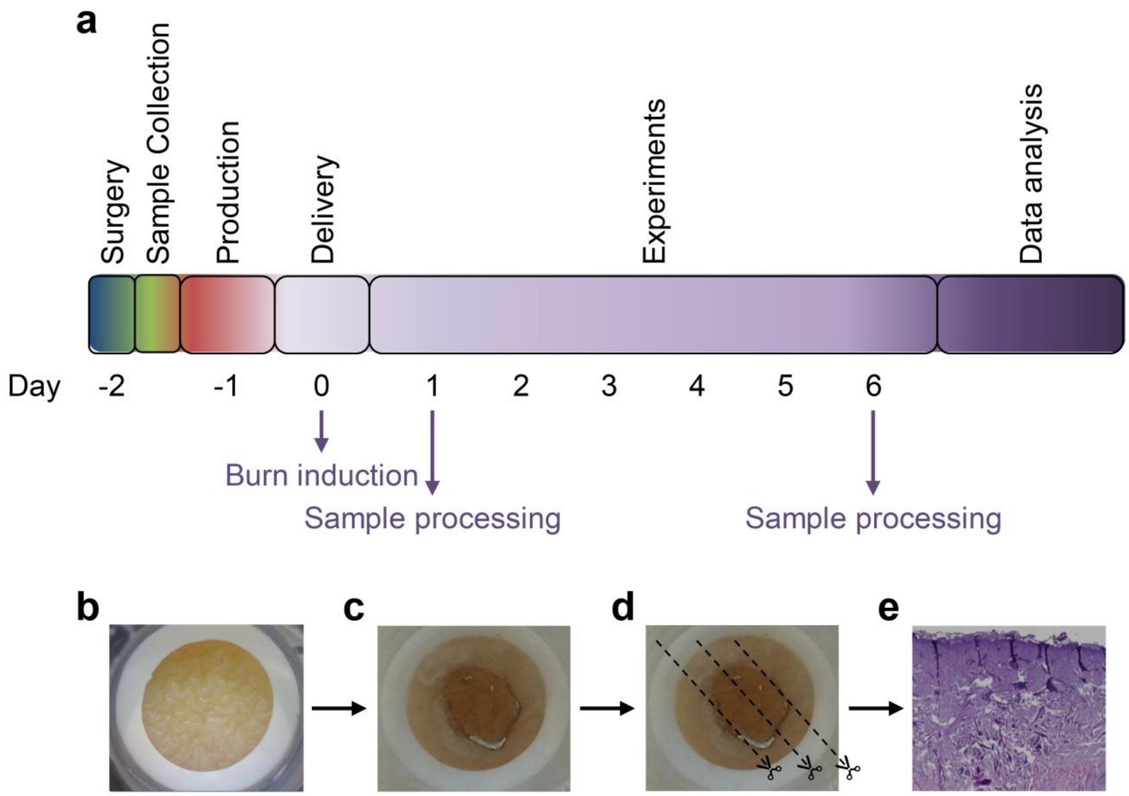

Fig. S1. Experimental setup for a native human skin burn wound infection model with *C. albicans*.

Surgery and sample production were done by a commercial provider (a). The prepared skin biopsies were embedded in a solid matrix within overhanging inserts (b). Culture medium was added to wells on arrival. All samples had a silicone ring surrounding the top keratinocyte layer of the skin, with the purpose of allowing improved topical application of compounds to the surface of the skin. Skin tissues were received on “day 0”, burnt (c), infected and depending on the experiment, supplemented with neutrophils. Samples were processed on day 1 and 6 post-infection (d) for histological staining (e; PAS, H&E in order to analyze wound area, wound depth, tissue injury), immunohistochemical staining (HMGB1 in order to analyze tissue viability) or immunofluorescent staining (NE in order to analyze neutrophil infiltration) and analysis of cytokine production (IL-1β, IL-6, IL-8).

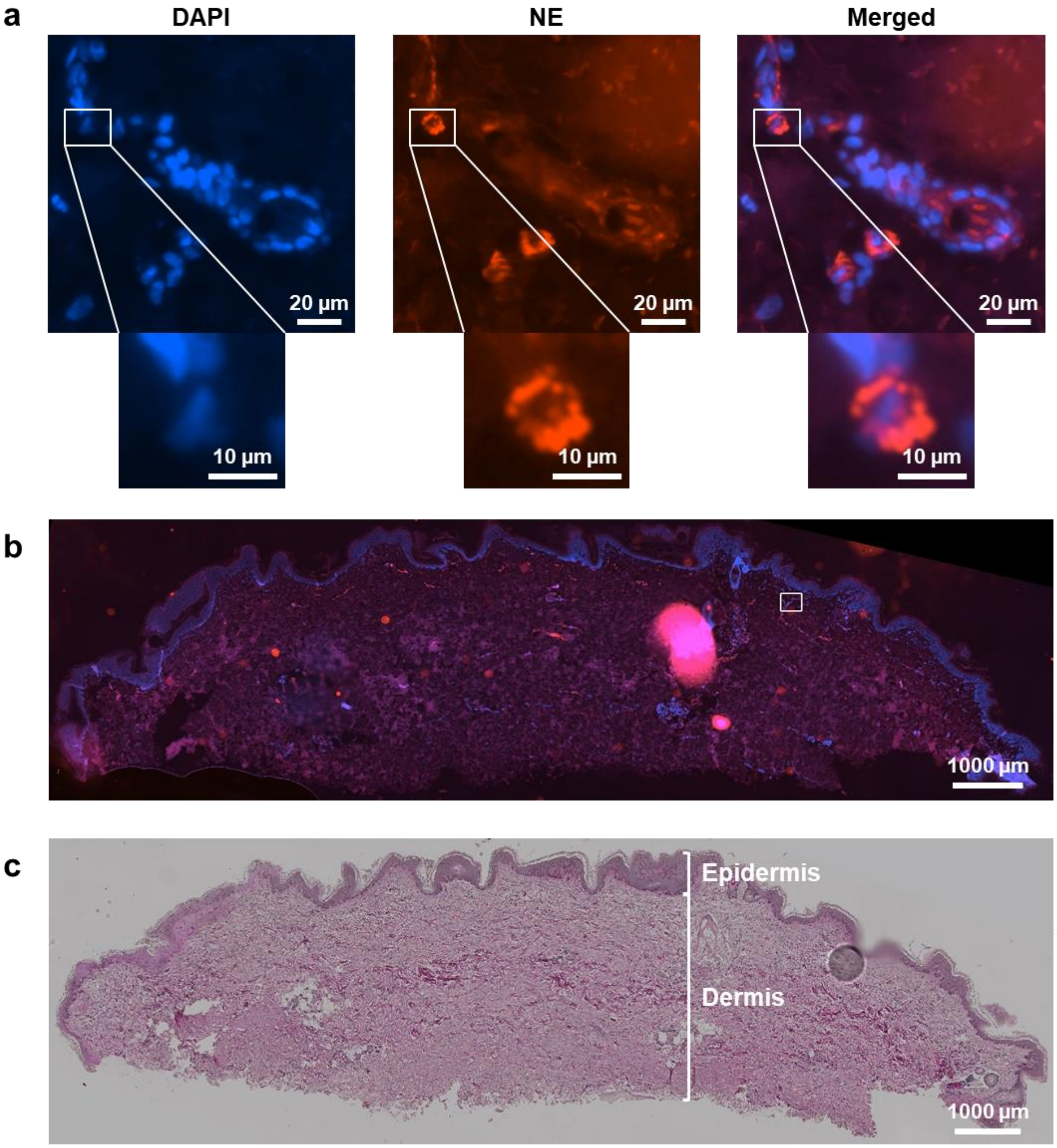

Fig. S2. Neutrophils are present in unburned control samples.

AF647-labelled immunofluorescence staining for neutrophil elastase (NE) shows a group of neutrophils present in dermal layers of an unburned skin tissue piece (a). A single neutrophil is about 10 µm in diameter. Left to right the columns show single channel images of DAPI staining the nucleus of cells, AF647-labelled NE marker and merged images. A whole scan of immunofluorescent stained tissue slice (b; DAPI, AF647) presents the region of interest

shown in Fig. S2A. A PAS-stained tissue slice (c) presents normal skin tissue structure with epidermal and dermal layers.

Samples are unburned, non-debrided, non-infected, without neutrophil supplementation, and fixed on day 6. Representative images are shown (N=5, n=6; N = number of donors, n = number of technical replicates).

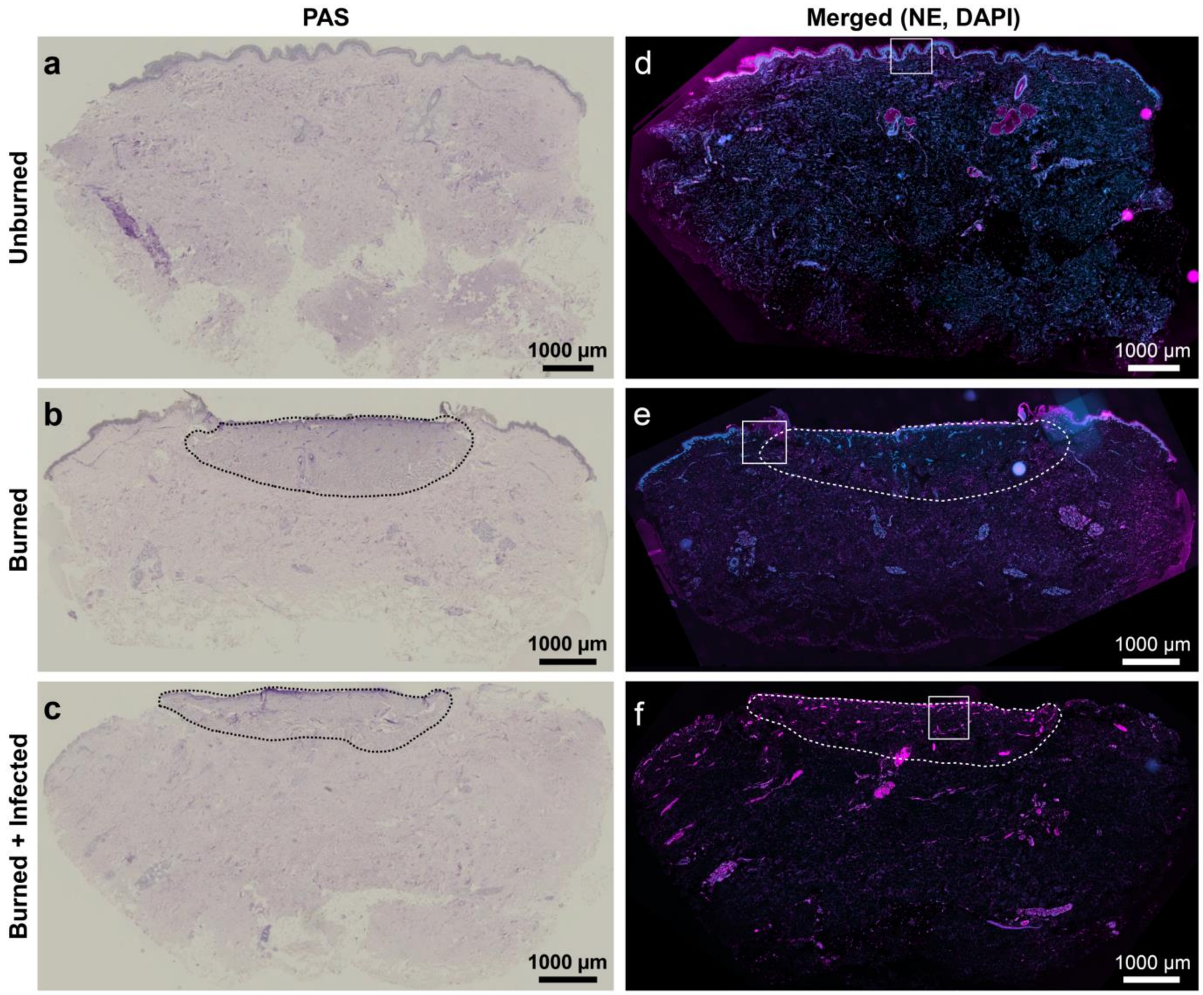

Fig. S3. Burning of skin tissue explants attracts neutrophils to the damaged area (Supplement to Fig. 5).

Unburned, burned, and burned-infected samples were stained applying PAS (left, a - c) for a histological overview, DAPI for localization of nuclei (blue) and AF647 labelled immunofluorescence staining (pink) for neutrophil elastase (NE) (right, d - f). Unburned control samples present with normal skin structure (a). The burned area has a darker purple color (b, c) than the surrounding tissue and *C. albicans* fungal cell walls stain magenta (c). Defined signals of NE localize tissue-resident inactive neutrophils, while diffuse signals indicate release of NE by activated neutrophils.

In unburned skin (d) neutrophils are present in dermal tissues whereas in burned (e) and burned-infected (f) skin neutrophils cluster at the epidermis.

All samples were fixed at day 6. Dotted lines present the burned area. Rectangles indicate the region enlarged in Fig. 5. For number of skin donors and technical replicates see Table S1. Representative images are shown.

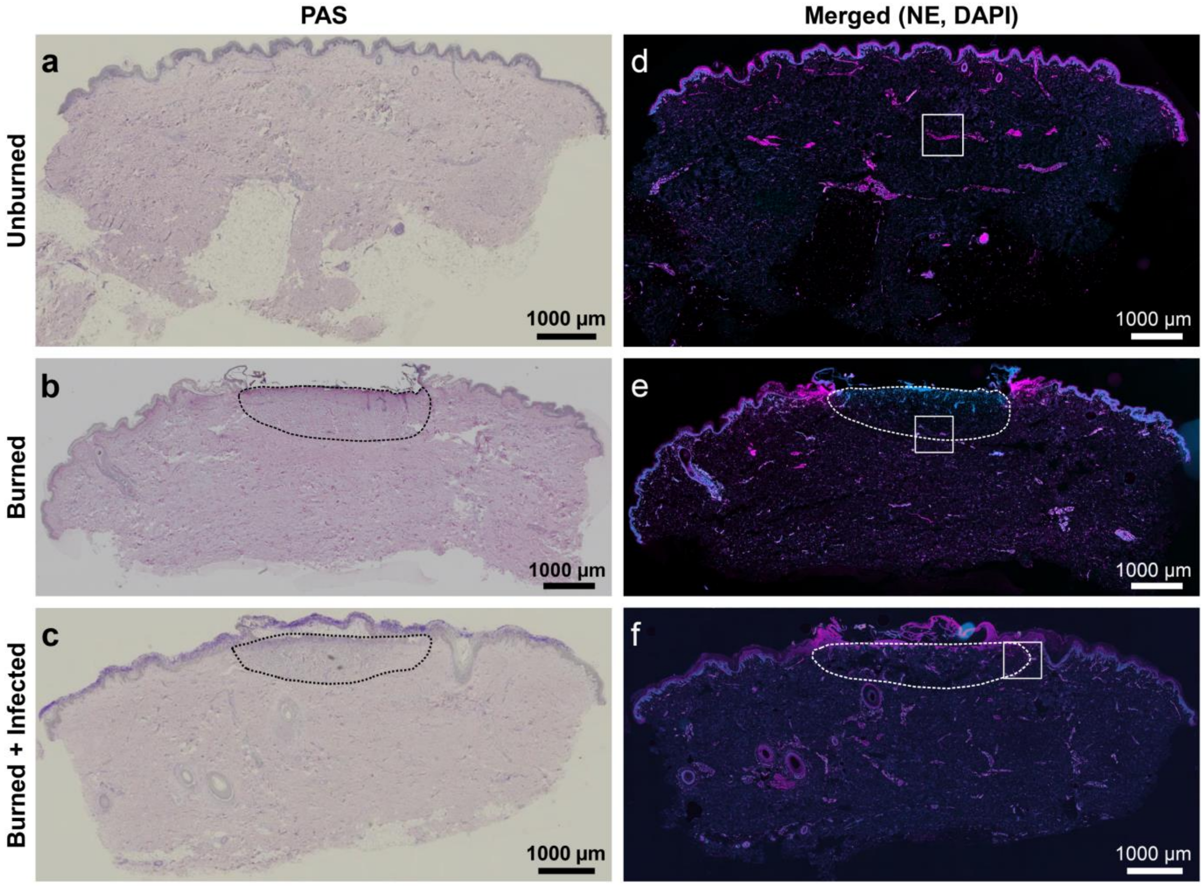

Fig. S4. Neutrophil supplementation to the media induces wound healing of burned skin tissue explants (Supplement to Fig. 6).

Unburned, burned, and burned-infected samples were stained applying PAS (left, a–c) for a histological overview, DAPI for localization of nuclei (blue) and AF647 labelled immunofluorescence staining (pink) for neutrophil elastase (NE) (right, d–f). Unburned control samples present with normal skin structure (a). The burned area has a darker purple color (b, c) than the surrounding tissue and *C. albicans* fungal cell walls stain magenta (c). Defined signals of NE localize tissue-resident inactive neutrophils, while diffuse signals indicate release of NE by activated neutrophils. Following neutrophil supplementation larger clusters of neutrophils are present in the lower dermal layers of

unburned skin tissue (d). Due to burning (e) or burning and infection (f) neutrophils migrate to the upper dermal layers and cluster at the site of damage and infection.

All samples were fixed at day 6. Dotted lines present the burned area whereas rectangles indicate the region enlarged in Fig. 6. For number of skin donors and technical replicates see Table S1. Representative images are shown.

Table S1. Number of donors and technical replicates throughout this study.

"N" is the number of donors and "n" the number of technical replicates.

**Burned**	**+**		**+**		**+**		**+**		**+**		**+**		**+**		**-**		**-**		**+**		**+**		**+**		**+**		**+**		**+**		**+**		**+**	
**Debrided**	-		-		-		+		+		+		+		-		-		-		-		-		-		+		+		+		+	
**Infected**	-		-		+		-		-		+		+		-		-		-		-		+		+		-		-		+		+	
**Neutrophil suppl.**	-		+		-		-		+		-		+		-		+		-		+		-		+		-		+		-		+	
**Day**	1		1		1		1		1		1		1		6		6		6		6		6		6		6		6		6		6	
	N	n	N	n	N	n	N	n	N	n	N	n	N	n	N	n	N	n	N	n	N	n	N	n	N	n	N	n	N	n	N	n	N	n
**Fig. 1**																			3	6														
**Fig. 2**	3	6																																
**Fig. 3**															5	6			2	1														
**Fig. 4a–d**																							3	3							4	4		
**Fig. 4e**																							1	2							1	2		
**Fig. 4f, g**																			3	23			1	3			2	9			3	13		
**Fig. 5**															5	6											3	3			4	4		
**Fig. 6a–c**																	3	3											3	4			3	4
**Fig. 6d, e**																			3	23	2	6	1	3	1	4	2	9	3	11	3	13	2	8
**Fig. 7a, b**	2	22			1	2	3	10	3	12	2	9	3	13					3	23			1	3			2	9	3	11	3	13	2	8
**Fig. 7c**	3	12	3	12			3	12	3	16	3	12	3	16					2	4	2	4					3	6	3	8	3	8	3	6
**Fig. 7d, e**	3	12	3	12			3	12	3	16	3	12	3	16					2	4	3	6					3	6	3	8	3	8	3	6
**Fig. S2**															5	6																		
**Fig. S3**															5	6											3	3			4	4		
**Fig. S4**																	3	3											3	4			3	4
